# Immune Regulation and Disulfidptosis in Atherosclerosis Influence Disease Progression and Therapy

**DOI:** 10.3390/biomedicines13040926

**Published:** 2025-04-09

**Authors:** Wei Lu, Zhidong Zhang, Gang Qiao, Gangqiang Zou, Guangfeng Li

**Affiliations:** Heart Center of Henan Provincial People’s Hospital, Central China Fuwai Hospital, Central China Fuwai Hospital of Zhengzhou University, Zhengzhou 451460, China; 15838127197@163.com (W.L.); gangqiao2004@126.com (G.Q.); m15138909798@163.com (G.Z.); 13673977521@163.com (G.L.)

**Keywords:** atherosclerosis, immune regulation, disulfidptosis, smooth muscle cells, inflammation, biomarkers, risk score model

## Abstract

**Background:** Atherosclerosis is a progressive and complex vascular pathology characterized by cellular heterogeneity, metabolic dysregulation, and chronic inflammation. Despite extensive research, the intricate molecular mechanisms underlying its development and progression remain incompletely understood. **Methods:** Single-cell RNA sequencing (scRNA-seq) was employed to conduct a comprehensive mapping of immune cell enrichment and interactions within atherosclerotic plaques, aiming to investigate the cellular and molecular complexities of these structures. This approach facilitated a deeper understanding of the heterogeneities present in smooth muscle cells, which were subsequently analyzed using pseudotime trajectory analysis to monitor the developmental trajectories of smooth muscle cell (SMC) subpopulations. An integrative bioinformatics approach, primarily utilizing Weighted Gene Co-expression Network Analysis (WGCNA) and machine learning techniques, identified Cathepsin C (CTSC), transforming growth factor beta-induced protein (TGFBI), and glia maturation factor-γ (GMFG) as critical biomarkers. A diagnostic risk score model was developed and rigorously tested through Receiver Operating Characteristic analysis. To illustrate the functional impact of CTSC on the regulation of plaque formation and SMC viability, both in vitro and in vivo experimental investigations were conducted. **Results:** An analysis revealed SMCs identified as the most prominent cellular type, exhibiting the highest density of disulfidptosis. Pseudotime trajectory analysis illuminated the dynamic activation pathways in SMCs, highlighting their significant role in plaque development and instability. Further characterization of macrophage subtypes demonstrated intercellular communication with SMCs, which exhibited specific signaling pathways, particularly between the proximal and core areas of plaques. The integrated diagnostic risk score model, which incorporates CTSC, TGFBI, and GMFG, proved to be highly accurate in distinguishing high-risk patients with elevated immune responses and systemic inflammation. Knockdown experiments of CTSC conducted in vitro revealed enhanced SMC survival rates, reduced oxidative stress, and inhibited apoptosis, while in vivo experiments confirmed a decrease in plaque burden and improvement in lipid profiles. **Conclusions:** This study emphasizes the significance of disulfidptosis in the development of atherosclerosis and identifies CTSC as a potential therapeutic target for stabilizing plaques by inhibiting SMC apoptosis and oxidative damage. Additionally, the risk score model serves as a valuable diagnostic tool for identifying high-risk patients and guiding precision treatment strategies.

## 1. Introduction

Atherosclerosis is a critical driver of the onset and progression of various cardiovascular diseases (CVDs), with coronary artery disease being the most prominent, as it can precipitate myocardial infarctions, commonly referred to as heart attacks. It is also closely associated with cerebrovascular diseases, which can result in strokes, and peripheral artery disease, which in severe cases may necessitate limb amputation. The burden of these diseases is substantial, as myocardial infarctions and strokes rank among the leading causes of death worldwide, surpassing fatalities attributed to cancer. In the United States, these diseases account for nearly 30 percent of all deaths, a statistic that aligns with global trends [1]. While several lipid-lowering therapies are available, the precise mechanisms driving the progression of atherosclerosis and the residual risk for cardiovascular events are incompletely understood.

Recently, a novel regulated cell death mechanism, termed disulfidptosis, has been proposed to be associated with a range of metabolic perturbations while also serving various roles in the context of anti-tumor immunity [2]. Disulfidptosis is characterized by alterations in cytoskeletal dynamics, in which glucose deprivation induces the aggregation of actin filaments and subsequent contraction of the cell. This process ultimately results in the detachment of the cytoskeleton from the cell membrane, thereby initiating cell death [3]. The phenomenon underscores the critical role of cellular metabolism in maintaining both structural integrity and function. Notably, disulfidptosis has demonstrated that metabolic perturbations in SLC7A11-activated cells can render them vulnerable to a deficiency of glucose and nicotinamide adenine dinucleotide phosphate (NADPH). This vulnerability presents an opportunity to exploit such metabolic dependencies and translate them into therapeutic strategies [4]. This discovery indicates that these cells may be particularly vulnerable to disruptions in their metabolic pathways. Researchers have identified an opportunity to leverage this dependence on glucose and NADPH, proposing several potential therapeutic strategies [5]. While much has been focused on cancer currently, such strategies open a broad platform to rewire metabolic vulnerabilities in other diseases driven by metabolic dysregulation.

Atherosclerosis is a chronic inflammatory disease characterized by the accumulation of lipids and immune cells within the arterial walls. This condition results in the thickening of arteries, a reduction in their elastic properties, and the formation of plaques on the inner surfaces of the arterial walls [6]. Metabolic syndromes are at the forefront of the initiation and development of atherosclerosis, prominently via inflammation, oxidative stress, and cellular energy metabolism [7,8,9]. While the exact role of disulfide stress towards atherosclerosis is poorly understood, it may impair cytoskeletal and membrane stability and contribute to its development [10]. Protein Disulfide Isomerase (PDI) is a crucial regulator of cellular homeostasis, particularly in vascular smooth muscle cells (VSMCs). This enzyme serves a dual function, modulating both cell survival and apoptosis in response to mechanical stress and advanced glycation end products (AGEs). Additionally, PDI is involved in complex biological processes such as VSMC proliferation for tissue repair and regeneration, alongside programmed cell death. The concurrent occurrence of these processes underscores PDI’s vital role in maintaining vascular health and its capacity to facilitate cellular adaptation to stress [11,12]. Targeting PDI activity presents a promising avenue for the treatment of pathological vascular remodeling. The inhibition of PDI results in the concurrent suppression of VSMC proliferation and apoptosis, thereby establishing it as a valid target for addressing vascular diseases. Additionally, PDI facilitates Nox1 activation through redox and oxidation interactions, which are crucial for VSMC migration and the progression of vascular disease. This mechanism involves disulfide crosslinks between PDI and the cytosolic component p47phox, which drive oxidative stress in VSMCs [13]. At the same time, dual PDI roles in oxidative stress and vascular remodeling remain especially important in atherosclerosis and in the context of other diabetic conditions [11]. Despite this, our understanding of the mechanisms through which disulfidptosis contributes to atherosclerosis remains limited, largely due to the insufficient knowledge surrounding both the phenomenon of disulfidptosis and the cellular processes involved in the progression of the disease. Consequently, there is a pressing need for further studies that elucidate the role of disulfidptosis in atherosclerosis and identify the cellular entities implicated in this complex process.

This study employed scRNA-seq to elucidate the immune cell composition in atherosclerotic plaques, revealing significant enrichment within the atherosclerotic core compared to proximal adjacent samples. The MuSiC algorithm and intercellular communication methods were utilized to evaluate the interactions among immune cells, highlighting the pivotal role of macrophages in the progression of atherosclerosis. A comprehensive analysis of macrophage diversity identified four distinct subtypes, each characterized by unique functional properties. Investigations into the pathways involved in macrophage development led to the identification of genes that delineate primary and related lineage differences among these subpopulations. Gene co-expression networks were constructed using WGCNA to highlight key genes for further exploration. Gene enrichment analyses provided valuable insights into the biological functions of these genes. Additionally, machine learning techniques identified potential biomarkers for atherosclerosis, facilitating the development of a highly accurate diagnostic model. A risk assessment system classified patients based on risk categories, while molecular and immune features were analyzed in relation to these categories.

## 2. Method

### 2.1. Data Acquisition

The scRNA-seq datasets utilized in this study were obtained from the Gene Expression Omnibus (GEO), specifically focusing on human atherosclerotic plaques. GSE155512 includes three plaques from distinct patients. Additionally, data from GSE159677, which comprised three atherosclerotic core plaques (AC) and three matched proximal adjacent plaques (PA), was employed to provide a comparative perspective. GSE131778 contributed eight individual plaques isolated from separate patients, thereby enriching and broadening the dataset’s spectrum. Further, additional bulk mRNA array datasets were extracted from various GEO and ArrayExpress repositories, specifically GSE120521, GSE41571, GSE163154, GSE28829, GSE43292, and GSE100927, to complement the single-cell data. To ensure comparability across datasets, GSE28829, GSE163154, and GSE43292 were harmonized using the Combat function in the ‘sva’ R package, effectively eliminating potential batch effects and ensuring comparability and reliability for subsequent downstream analyses. After excluding three outlier samples, adjustments were made for a test set comprising 60 stable plaques and 73 unstable plaques. For validation purposes, GSE120521, GSE41571, GSE100927, and E-MTAB-2055 were selected. The pre-processing and normalization of raw data were conducted using the Robust Multiarray Average (RMA) algorithm available in the ‘affy’ R package.

### 2.2. scRNA-seq Data Processing

A package called ‘Seurat’ processed the input scRNA-seq data by retaining genes expressed in more than three cells and applying additional filters to the cells, restricting the number of detected genes to between 200 and 5000, with an RNA count below 25,000 or above 15% mitochondrial gene content. This preprocessing resulted in data from 68,064 cells available for downstream analysis. Data normalization and scaling were conducted using the ‘NormalizeData’ and ‘ScaleData’ functions, while the top 3000 variable genes were identified through ‘FindVariableFeatures’. The ‘RunHarmony’ function was employed to address batch effects present between samples, ensuring dataset consistency. Principal Component Analysis (PCA) was performed to determine anchor points for dataset alignment, thereby reducing dimensionality. The t-SNE algorithm was subsequently applied to visualize cell clusters based on the top 15 principal components, providing intuitive insights into cellular distributions and relationships. Using the functions ‘FindNeighbors’ and ‘FindClusters’, a total of 17 distinct clusters were identified, with the resolution parameter set to 0.4 to achieve a reasonable granularity in the clustering analysis. Some clusters were characterized by cell markers identified using the COSG R package, which facilitated the precise definition of cluster identities. In this context, parameters were specifically tuned, including a mean (mu) of 10 and a user-defined number (n_genes_user = 50) of 50 genes to refine marker selection and cluster annotation.

### 2.3. Cell Communication Analysis

Communication between the PA and AC groups was analyzed using the CellChat R package [14]. Ligand–receptor interaction data were sourced from the CellChatDB.human database, serving as a comprehensive reference for communication pathways. Default parameters were employed for the communication analysis, and the functions mergeCellChat and compareInteractions were utilized to facilitate a comparative evaluation of interactions between the two groups. To illustrate differences in the relative strength of interactions and the frequency across various cell types, the netVisual_circle function provided an intuitive graphical representation of the cellular communication network. Additionally, to show and compare the expression distributions of key signaling genes between the groups, the netVisual_bubble function was adopted to detail the variation in ligand-receptor activity when comparing PA and AC conditions.

### 2.4. Disulfidptosis Score Calculation

Cell disulfidptosis scores were evaluated using architectures based on ‘AUCell’, ‘UCell’, ‘singscore’, and ‘ssgsea’. These approaches employed a curated set of 14 key genes associated with disulfidptosis to provide comprehensive and well-founded estimates of cellular activity related to disulfidptosis. Violin plots were utilized to visualize the differences in disulfidptosis scores across various cell types and algorithms.

### 2.5. Trajectory Analysis

The trajectory analysis conducted with the Monocle2 package was proposed to assess the differentiation route of pertinent clusters [15]. The ‘subset’ command in Seurat was utilized to extract the relevant cell clusters. Subsequently, a CellDataSet object was generated using the ‘newCellDataSet’ function from the Monocle2 framework. Low-quality cells and genes were eliminated through the ‘detectGenes’ and ‘subset’ functions. Genes exhibiting differential expression throughout the trajectory were identified using the ‘differentialGeneTest’ function. The ‘DDRTree’ method was employed for dimensionality reduction, and visualizations were created using ‘plot cell trajectory’, ‘plot genes in pseudotime’, and ‘plot genes branched heatmap’.

### 2.6. Phenotype Score Estimation

Metrics related to phenotype, including cholesterol efflux, ferroptosis, angiogenesis, phagocytosis, autophagy, lysosomal function, hypoxic conditions, inflammatory responses, and endoplasmic reticulum stress, were obtained from the Molecular Signatures Database (MSigDB). The AUCell algorithm was utilized to compute these metrics across various groups, with the results visually represented through violin plots. To annotate cell types in Bulk RNA-seq, the MuSiC deconvolution algorithm was employed to assess the relative proportions of different cell types within the RNA-seq data from GSE100927. This method leverages gene expression profiles specific to cell types, derived from scRNA-seq data, to ascertain cell abundance in bulk samples.

### 2.7. WGCNA Analysis

WGCNA was conducted to construct gene co-expression networks from GSE100927 using the ‘WGCNA’ R package. Genes exhibiting the top 25% variance were selected for analysis, and genes with missing values were excluded using the ‘goodSamplesGenes’ function. A visual assessment was performed to determine an appropriate soft threshold for calculating adjacency, which subsequently transformed the adjacency matrix into a topological overlap matrix. Hierarchical clustering analysis facilitated the identification of gene modules, followed by an examination of module eigengenes (MEs) in relation to clinical characteristics. Modules demonstrating the highest correlation with SMCs-related genes were prioritized for further analysis.

### 2.8. Machine Learning Models for Feature Selection

Several machine learning approaches, including Least Absolute Shrinkage and Selection Operator (LASSO), Support Vector Machine Recursive Feature Elimination (SVM-RFE), and Random Forest (RF) models, were implemented for feature selection purposes. The analysis was conducted using the R packages ‘glmnet’, ‘e1071’, ‘caret’, and ‘Boruta’, ensuring a robust and comprehensive identification of significant features. The LASSO model was optimized for the penalty parameter (λ) through five-fold cross-validation, facilitating precise selection of relevant predictors. SMCs-related genes deemed significant according to the Boruta algorithm (300 iterations with a strict *p*-value threshold of <0.01) were further refined. These genes were subsequently assessed using the SVM-RFE and RF models to identify the most informative features that enhanced the accuracy of the interpretations. Cross-validation was employed to prevent overfitting. The final predictive genes were determined by taking the intersection of the results from each of the models.

### 2.9. Risk Model Construction and Assessment

Utilizing the identified SMCs-related genes, a risk model was developed with the ‘rms’ R package. A nomogram was created to estimate individual risk scores, and calibration was performed using a calibration curve. To assess the clinical utility of the nomogram, decision curve analysis (DCA) was conducted with the ‘ggDCA’ package. Patients with a median risk score were classified into high-risk and low-risk groups, and the model’s predictive efficacy was evaluated through Receiver Operating Characteristic (ROC) analysis.

### 2.10. Enrichment Analysis

ClusterProfiler—An R package was used to perform enrichment analysis incorporating Kyoto Encyclopedia of Genes and Genomes (KEGG) and Gene Ontology (GO) analyses to address processes of biological (BP), molecular functions (MF), and cellular components (CC) [16]. All statistically significantly defined features received a threshold of *p*-value < 0.05. GSVA analysis was further conducted in quantifying activities of biological pathways using the Hallmark gene sets from MSigDB [17]. The Limma package established the significance of differential pathway activities by checking absolute t-values above 2. Furthermore, a GSEA was performed when testing for differences in pathway activities.

### 2.11. Atherosclerotic Immunity Assessment

Algorithms such as ssGSEA, MCPcounter, xCell, ABIS, and ESTIMATE were employed to assess levels of immune infiltration and define immune cell proportions in each sample. The Wilcoxon rank-sum test was utilized to compare immune infiltration levels between the groups, serving as a statistical framework for evaluating differences in immune cell proportions. Differences in immune infiltration were subsequently visualized using heat maps, which provided an intuitive representation of the distribution patterns of immune cell subsets across the samples. Additionally, a comprehensive analysis of immune checkpoint gene expression was conducted to compare differences between groups, highlighting the potential variations in their regulatory roles within the immune microenvironment.

### 2.12. Construction of Atherosclerosis SMC Model and Lentiviral Transfection

The A7r5 cell line of rat thoracic aortic SMCs was obtained from the Cell Bank of the Chinese Academy of Sciences (Shanghai, China) and cultured in Dulbecco’s Modified Eagle Medium supplemented with 10% fetal bovine serum. The cells were maintained at 37 °C in a humidified atmosphere containing 5% CO_2_. In this study, an in vitro model of atherosclerosis was established by treating A7r5 SMCs with 50 µg/mL of oxidized low-density lipoprotein for 24 to 48 h. For gene knockdown experiments, lentiviral vectors targeting CTSC and control vectors were procured from Genechem (Shanghai, China). The cells were divided into three experimental groups: (1) control group, (2) atherosclerosis model group with an empty vector, and (3) atherosclerosis model group with CTSC knockdown. Lentiviral transfection was conducted at a multiplicity of infection (MOI) of 3 in the presence of 5 µg/mL polybrene to enhance transduction efficiency. After 48 h, real-time quantitative polymerase chain reaction analysis confirmed siRNA-mediated inhibition of the CTSC gene.

### 2.13. Apoptosis Detection by Flow Cytometry

The cells were collected and processed for apoptosis analysis using Annexin V-FITC and propidium iodide (PI) staining in accordance with the manufacturer’s protocol. Following a 15 min incubation in the dark at room temperature, the samples were analyzed using flow cytometry on the CytoFLEX system. The fractions of apoptotic cells were determined by calculating the percentages of the early and late apoptotic subsets within the cell population, thereby providing detailed information on the dynamics of cell death.

### 2.14. Reactive Oxygen Species (ROS) Detection

To detect ROS, the culture medium was discarded, and the cells were washed three times with either phosphate-buffered saline (PBS) or a serum-free medium. Dihydroethidium (DHE) from KeyGEN BioTECH was prepared in dimethyl sulfoxide (DMSO) to create a stock solution of 5 mM, which was subsequently diluted to a 1:500 concentration in PBS or serum-free medium for the staining procedure. The cells were treated with the working solution at 37 °C for 30 min, ensuring protection from light. After the washes, Hoechst 33342 from Solarbio was used to stain the nuclei, with cells incubated in darkness for 20–30 min. A fluorescence microscope (IX51, Olympus or Eclipse Ci-L, Nikon, Tokyo, Japan) was employed to image the stained cells, with the assessment of ROS levels based on the intensity of red fluorescence, while Hoechst served as a nuclear counterstain.

### 2.15. Cell Counting Kit-8 (CCK-8) Assay for Cell Viability

Cell viability was assessed using the CCK-8 assay (Sigma, Livonia, MI, USA). A7r5 cells were seeded in 96-well plates, and following treatment, 10 µL of CCK-8 reagent was added to each well. The plates were then incubated at 37 °C for 2 h, after which absorbance measurements were recorded at 450 nm using a microplate reader. The results for cell viability were expressed as a percentage relative to the control group.

### 2.16. Animal Treatment, Atherosclerosis Induction, and CTSC Knockdown

Thirty male Sprague Dawley (SD) rats, aged between 6 and 8 weeks and weighing between 180 g and 200 g, were obtained from Beijing Vital River Laboratory Animal Technology. The rats were randomly assigned to three groups: a control group, an atherosclerosis group with solvent control, and an atherosclerosis group for CTSC knockdown. To induce atherosclerosis, the experimental group was fed a high-fat diet containing 1% cholesterol, 0.5% sodium cholate, and 10% lard for 12 weeks, while the control group received a standard chow diet. Additionally, each rat was administered a single high-dose vitamin D3 injection at 600,000 IU/kg via intraperitoneal injection to facilitate the development of atherosclerosis. CTSC knockdown was achieved by injecting an adeno-associated virus (AAV) vector specifically targeting CTSC into the tail vein every 14 days. In contrast, the solvent control group received an equal volume of viral vehicle. The efficiency of CTSC knockdown was validated through RT-qPCR analysis.

### 2.17. Blood Lipid Analysis

Blood samples were collected twice at the end of the 12-week study period after a 12 h fasting period. An automatic biochemical analyzer (Roche) was utilized to assess the serum levels of total cholesterol (TC), low-density lipoprotein cholesterol (LDL-C), and triglycerides (TGs).

### 2.18. Histopathological Examination

Following the 12-week study, rats were anesthetized with 2–3% isoflurane administered via inhalation. The thoracic cavity was accessed, and phosphate-buffered saline (PBS) was perfused through the aorta to remove any residual blood. Both the thoracic and abdominal sections of the aorta were carefully dissected and cleaned using a stereomicroscope. Subsequently, segments of the aorta were prepared for histological analysis and staining.

### 2.19. RT-qPCR

Total RNA was extracted from peripheral blood samples obtained from rats and from A7r5 cells using Trizol reagent sourced from Invitrogen, USA. Following this extraction process, the RNA underwent reverse transcription to synthesize complementary DNA, utilizing the RevertAid First Strand cDNA Synthesis Kit. To quantify the resulting cDNA, a quantitative reverse transcription–polymerase chain reaction was performed using the Mx3000P QPCR System manufactured by Stratagene in La Jolla, CA, USA.

### 2.20. Statistical Analysis

Data analysis was conducted using R software. To evaluate the relationships among continuous variables, Spearman’s correlation analysis was employed. The Wilcoxon rank-sum test or a two-tailed t-test was used for group comparisons involving continuous variables. Chi-square tests were applied to categorical data. *p*-values greater than 0.05 were considered non-significant (ns), while *p*-values less than 0.05, 0.01, 0.001, and 0.0001 were regarded as significant, denoted by * *p*, ** *p*, *** *p*, and **** *p*, respectively.

## 3. Result

### 3.1. Immune Cell Composition and Subtypes in Atherosclerotic Plaques Revealed by scRNA-Seq Analysis

**Figure 1:**
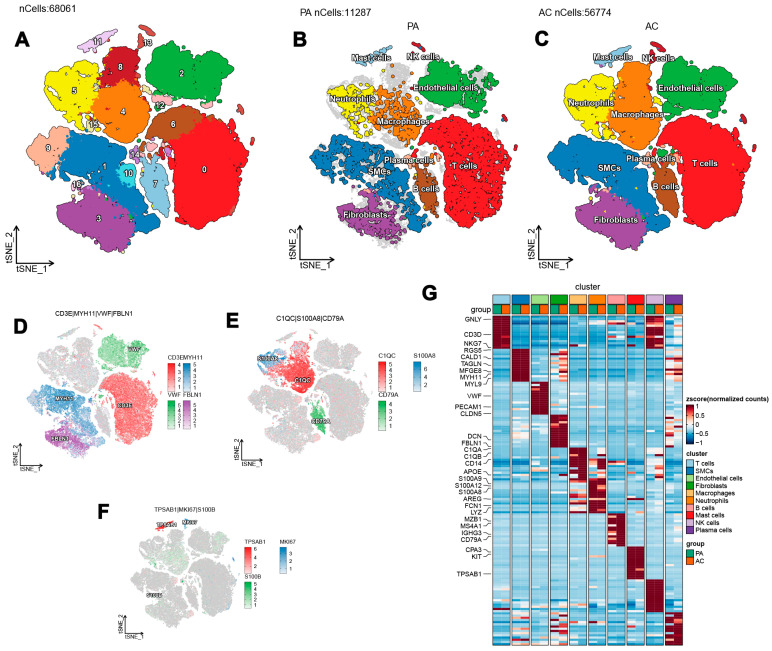
Single-cell RNA sequencing analysis of immune cells in atherosclerotic plaques. (**A**) Distribution of cell clusters in the combined dataset. (**B**) Cell cluster distribution in the PA group. (**C**) Cell cluster distribution in the AC group. (**D**–**F**) Cell counts and marker genes for each immune cell subtype. (**G**) Top ten characteristic genes for each cell type in PA and AC groups.

The analysis utilizing scRNA-seq was conducted to systematically evaluate the immune cell composition within atherosclerotic plaques. After implementing extensive quality control measures, a total of 68,061 high-quality cells were selected, comprising 12,287 cells from PA plaque samples and 56,774 cells from AC plaque samples. These selected cells were deemed suitable for subsequent analyses. The overall arrangement of cell clusters within the dataset is illustrated in Figure 1A, with distinct distributions for PA and AC groups depicted in Figure 1B,C, respectively. The distribution of cells across various clusters was quantified, resulting in the identification of 10 immune cell subtypes in the integrated dataset. This classification included T cells (*n* = 19,751), characterized by CD3E expression; smooth muscle cells (SMCs, *n* = 12,571), indicated by MYH11; endothelial cells (*n* = 9304), identified by VWF; macrophages (*n* = 9233), associated with C1QC; and fibroblasts (*n* = 6962), expressing FBLN1. Additional subtypes included neutrophils (*n* = 5188), indicated by S100A8; B cells (*n* = 3031), expressing CD79A; mast cells (*n* = 865), defined by TPSAB1; natural killer (NK) cells (*n* = 714), identified by MK167; and plasma cells (*n* = 406), associated with S100B. These results are visually summarized in Figure 1D–F. Furthermore, the top ten signature genes associated with each immune cell type within the PA and AC groups were identified and are represented in Figure 1G. The expression patterns of these hallmark genes for the ten cell types in the integrated dataset further corroborated their classification, as shown in Figure S1.

### 3.2. Intercellular Communication and Signaling Pathways of SMCs in Atherosclerosis

**Figure 2:**
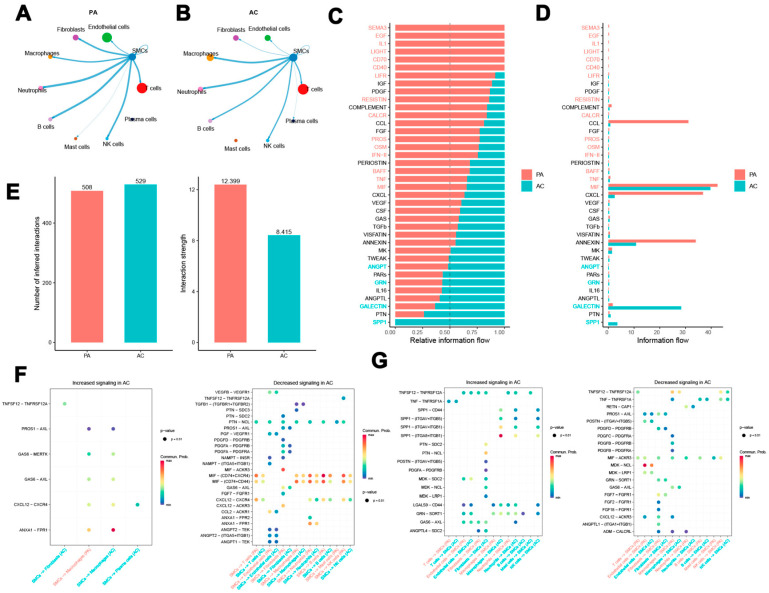
Intercellular communication analysis results. (**A**,**B**) Interactions between SMCs and various cell types in the PA (**A**) and AC (**B**) groups are illustrated. A thicker line denotes a more robust interaction, while a larger circular dot signifies a greater quantity of interactions. (**C**) Active signaling pathways related to SMCs in both groups. (**D**) MIF signaling pathway activation in both groups. (**E**) Key ligand–receptor pairs between SMCs and other cell types. (**F**,**G**) Upregulated and downregulated signaling pathways involving SMCs in the AC group.

In this research, the MuSiC algorithm was employed to infer the distribution of cell subpopulations from the GSE100927 bulk transcriptome dataset, utilizing single-cell data as a reference. The analysis revealed a pronounced expression of SMCs within the AC group, prompting their isolation for further investigation. An assessment of intercellular communication indicated that SMCs in the PA group exhibited notable interaction strength with various cell types, including endothelial cells, fibroblasts, macrophages, neutrophils, natural killer (NK) cells, T cells, and with each other. Significant connectivity was particularly evident between SMCs and B cells, T cells, and neutrophils. Endothelial cells, fibroblasts, SMCs, and T cells demonstrated a substantial number of interactions with SMCs (Figure 2A). Similarly, in the AC group, SMCs displayed strong interactions, particularly with macrophages, neutrophils, B cells, NK cells, T cells, and among themselves (Figure 2B). In contrast, SMCs in the PA group exhibited greater interaction strength but engaged in fewer interactions with other cell types compared to those in the AC group (Figure 2E). These findings suggest that SMCs may play a critical role in the development of both PA and AC. The analysis of variations in interaction strength among the groups revealed specific signaling pathways associated with SMCs. In the PA group, pathways such as SEMA3, EGF, IL1, LIGHT, CD70, CD40, LIFR, RESISTIN, CALCR, PROS, OSM, IFN-II, BAFF, TNF, and MIF demonstrated increased activity, with CCL, CXCL, MIF, and ANNEXIN exhibiting particularly pronounced activation. Conversely, the AC group displayed activation of ANGPT, GRN, GALECTIN, and SPP1 pathways (Figure 2C). Notably, CCL, CXCL, and ANNEXIN were exclusive to the PA group, while SPP1 and GALECTIN were specific to the AC group. Intriguingly, MIF showed significant activation in both cohorts (Figure 2D).

Key ligand–receptor interactions between SMCs and various other cell types were further examined (Figure 2E). In the atherosclerotic group, SMCs acted as ligand cells, enhancing interactions such as TNFSF12-TNFRSF12A with fibroblasts, as well as CXCL12-CXCR4, GAS6-AXL, GAS6-MERTK, and PROS1-AXL with macrophages, which were observed in both the AC and PA groups. Conversely, SMCs also functioned as ligand cells by attenuating several pathways with other cellular entities, including MIF-(CD74+CXCR4) and MIF-(CD74+CD44), seemingly engaging with a variety of cell types (Figure 2F). When acting as receptor cells in the AC group, SMCs intensified pathways such as LGALS9-CD44, GRN-SORT1, TNFSF12-TNFRSF12A, TNF-TNFRSF1A, SPP1-CD44, SPP1-(ITGAV+ITGB5), SPP1-(ITGAV+ITGB1), SPP1-(ITGA8+ITGB1), and MDK-SDC2. However, interactions with other cell types were associated with the inhibition of pathways, including TNFSF12-TNFRSF12A, TNF-TNFRSF1A, RETN-CAP1, MIF-ACKR3, PROS1-AXL, CXCL12-ACKR3, and ADM-CALCRL (Figure 2G). These results underscore the complex intercellular communication occurring between SMCs and other cell types in the context of atherosclerosis, highlighting the necessity for further validation of these findings.

### 3.3. Disulfidptosis and SMC Subcluster Characterization in Atherosclerosis

**Figure 3:**
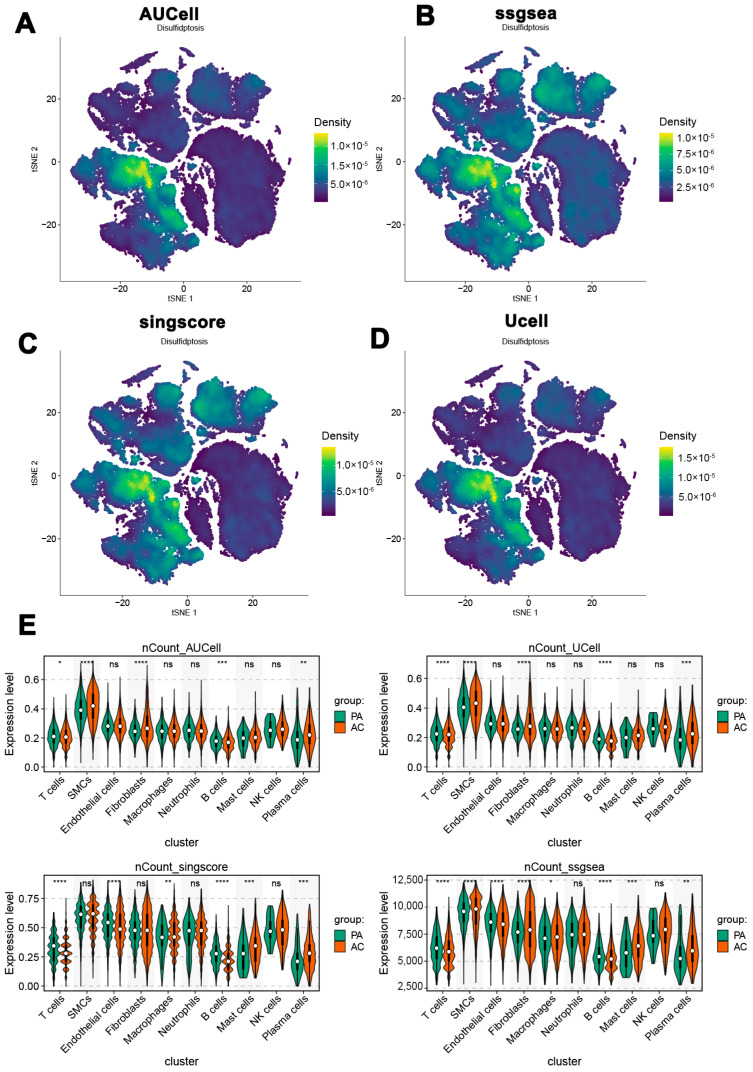
Distribution of disulfidptosis scores across cell types in atherosclerosis patients. (**A**–**D**) Density of disulfidptosis in SMCs detected by four different algorithms. (**E**) Expression of disulfidptosis-related genes in PA and AC groups. * *p* < 0.05, ** *p* < 0.01, *** *p* < 0.001, **** *p* < 0.0001.

**Figure 4:**
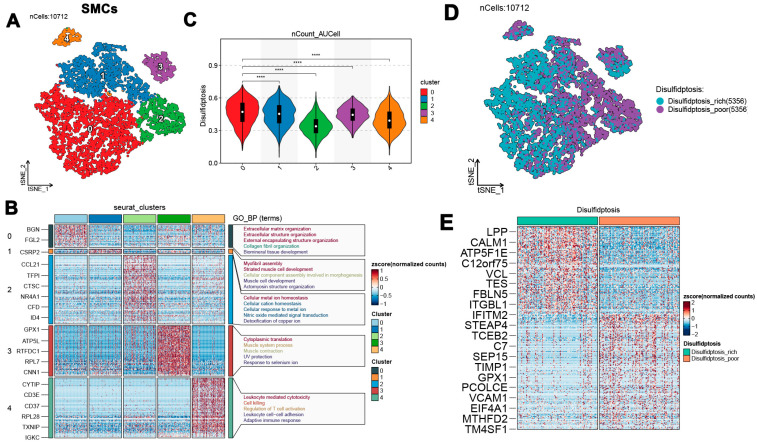
Characterization of smooth muscle cell (SMCs) subpopulations. (**A**) Identification of five distinct SMC subclusters based on signature genes. (**B**) Enrichment analysis of pathways for each SMC subcluster. (**C**) Distribution of disulfidptosis scores across SMCs subclusters. (**D**) Distribution of disulfidptosis-rich and disulfidptosis-poor groups (**E**) Characteristic genes of disulfidptosis-rich and disulfidptosis-poor groups. **** *p* < 0.0001.

Four algorithms—‘AUCell’, ‘ssGSEA’, ‘singscore’, and ‘UCell’—were utilized to analyze the distribution of disulfidptosis across cell types in atherosclerosis patients within the combined dataset. Notably, SMCs exhibited the highest density of disulfidptosis across all four methods (Figure 3A–D). Furthermore, disulfidptosis-related genes demonstrated substantial expression in both the PA and AC groups, as indicated by these algorithms (Figure 3E). These findings suggest a potential association between SMC activation and disulfidptosis in the context of atherosclerosis. A detailed analysis of SMCs within the combined dataset identified five distinct subclusters based on their signature gene expression profiles (Figure 4A). Functional enrichment analysis using Gene Ontology Biological Processes (GO_BP) was performed for the signature genes of each cluster. Cluster 0, characterized by genes such as BGN and FGL2, was enriched in pathways related to extracellular matrix organization, collagen fibril organization, and biomineral tissue development. Cluster 1, marked by CSRP2, was associated with pathways related to myofibril assembly, muscle cell development, and actomyosin structure organization. Cluster 2, defined by genes such as TFPI, CTSC, and NR4A1, was enriched in pathways related to cellular metal ion homeostasis, nitric oxide-mediated signal transduction, and copper ion detoxification. Cluster 3, featuring genes like GPX1 and ATP5L, exhibited pathways involved in cytoplasmic translation, muscle contraction, and UV protection. Finally, Cluster 4, containing genes such as CYTIP, CD3E, and TXNIP, was linked to leukocyte-mediated cytotoxicity, T-cell activation regulation, and adaptive immune responses (Figure 4B).

Disulfidptosis scores for each cluster were calculated using the AUCell algorithm. Violin plot analysis indicated that clusters 0 and 1 exhibited relatively high disulfidptosis scores, while clusters 2, 3, and 4 showed lower scores (Figure 4C). Based on the median disulfidptosis score, SMCs were categorized into disulfidptosis-rich and disulfidptosis-poor groups (Figure 4D). Heatmap analysis identified characteristic genes for these groups: disulfidptosis-rich SMCs were enriched in genes such as LPP, CALM1, and VCL, whereas disulfidptosis-poor SMCs were characterized by genes including STEAP4, TCEB2, and VCAM1 (Figure 4E). Phenotypic scores were calculated in greater detail and compared across the two groups. The disulfidptosis-rich group exhibited increased scores across nearly all phenotypic categories, including angiogenesis, phagocytosis, autophagy, hypoxia, the acute inflammatory response, and endoplasmic reticulum stress. Conversely, the disulfidptosis-poor group reported higher scores related to cholesterol efflux and lysosomal pathways (Figure S2). This analysis highlights the complex role of disulfidptosis within SMC subclusters, suggesting potential implications for the pathology of atherosclerosis and underscoring the need for further research.

### 3.4. Pseudotime Analysis of SMC Development and Disulfidptosis in Atherosclerosis

**Figure 5:**
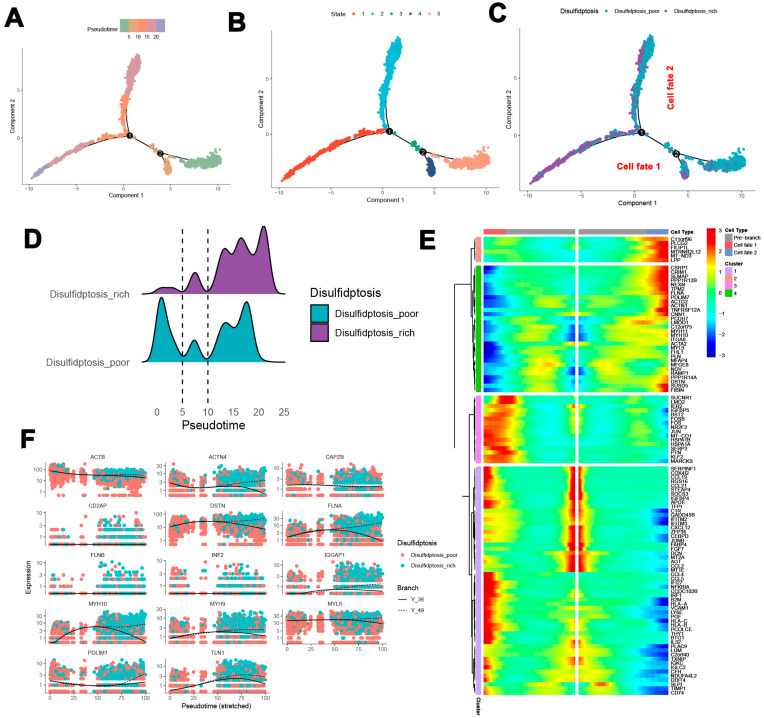
Pseudotime developmental trajectory analysis of SMCs. (**A**) Pseudotemporal trajectory of SMCs over time. (**B**) Classification of SMC states at different time points. (**C**) Primary developmental trajectory showing SMC differentiation. (**D**) Activation trends in disulfidptosis-high and disulfidptosis-poor groups. (**E**) Heatmap of characteristic gene distribution in each group and cell cluster. (**F**) Expression of selected characteristic genes in the two groups.

To enhance the understanding of immune dynamics, we analyzed pseudotime developmental trajectories of SMCs to determine the optimal curve that illustrates cell differentiation and development. This examination provided valuable insights into the lineage framework of SMCs in relation to atherosclerotic plaques. Figure 5A presents the pseudotemporal trajectory of SMCs over time, revealing two prominent branch points. Based on distinct time intervals, SMCs were classified into five states (Figure 5B), illustrating a primary developmental pathway in which SMCs diverge into two distinct fates over time (Figure 5C). Further pseudotemporal analysis was conducted for both the disulfidptosis-poor and disulfidptosis-rich groups. The results indicated that the disulfidptosis-rich group exhibited a clear activation trend as time progressed. In contrast, the disulfidptosis-poor group displayed high activation at the initial stage, followed by a decrease and then a subsequent increase in activation (Figure 5D). A heatmap depicting the distribution of characteristic genes across each group and cell cluster is presented in Figure 5E. Finally, the expression levels of 14 characteristic genes in both groups are illustrated in Figure 5F.

### 3.5. Gene Co-Expression Network and Functional Analysis of Key Atherosclerosis-Associated Genes

**Figure 6:**
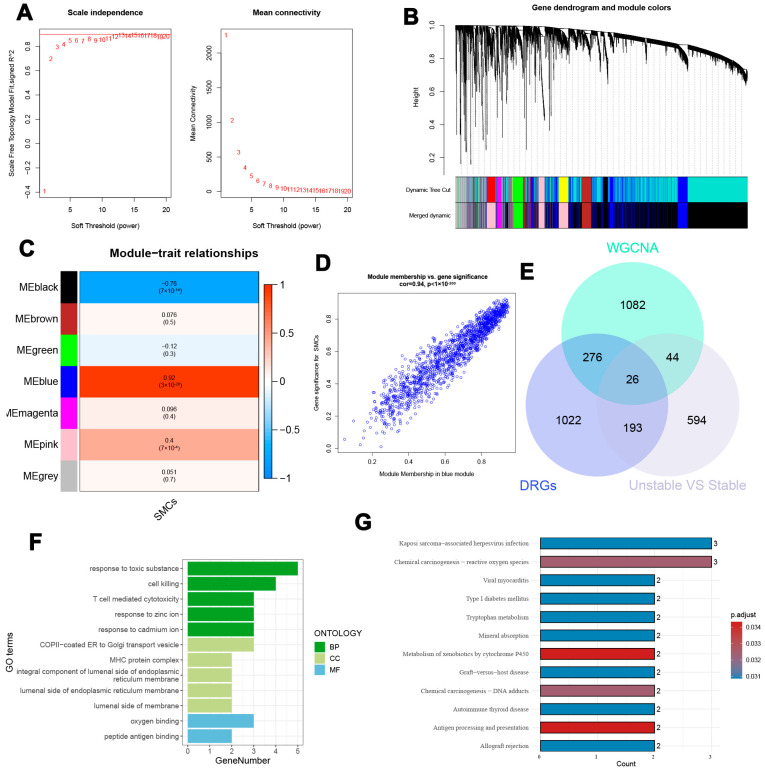
Identification and enrichment analysis of characteristic genes. (**A**) In WGCNA, an ideal soft threshold is established to calculate adjacency. (**B**) The co-expression modules are categorized, and the resultant dendrogram is presented. (**C**) Identification of nine different gene co-expression modules, each denoted by a distinctive color in the dendrogram. (**D**) Number of genes associated with the blue module significantly correlated with SMCs. (**E**) Intersection of 26 characteristic genes identified from the analysis. (**F**) Gene Ontology (GO) analysis of the identified genes. (**G**) Kyoto Encyclopedia of Genes and Genomes (KEGG) pathway analysis results.

The WGCNA algorithm was employed to construct a gene co-expression network by integrating datasets GSE28829, GSE163154, and GSE43292. An optimal soft-thresholding power β of 8 was selected to enhance the hierarchical clustering of samples, resulting in the identification of nine distinct gene co-expression modules, each represented by a unique color in the dendrogram, as illustrated in Figure 6A–C. The blue module exhibited a strong correlation (R = 0.92) with SMCs, leading to the identification of 1428 genes for further analysis (Figure 6C,D). Furthermore, a significant positive relationship was observed between the blue module and its associated genes. During the pseudotime analysis, a total of 1517 genes were identified, with 857 hallmark genes confirmed through comparisons of stable and unstable plaques from E-MTAB-2055. By integrating data from WGCNA, pseudotime analysis, and the hallmark genes derived from E-MTAB-2055, we identified 26 unique genes, as depicted in Figure 6E. Subsequent enrichment analyses were performed to investigate the biological roles of these 26 genes. GO analysis revealed their involvement in various biological processes, including responses to toxic agents, induction of cell death, mediation of T-cell cytotoxicity, and reactions to zinc and cadmium ions. In terms of CC, these genes were primarily associated with functions related to the cell membrane. The MF analysis indicated that these genes are involved in both oxygen and peptide antigen binding, as shown in Figure 6F. Additionally, KEGG pathway analysis highlighted the participation of these genes in diverse biological activities, such as immune responses, infections, and metabolic and genetic alterations, underscoring their potential significance in various diseases and physiological conditions, as illustrated in Figure 6G.

### 3.6. Screening of Characteristic Genes Based on Machine Learning

**Figure 7:**
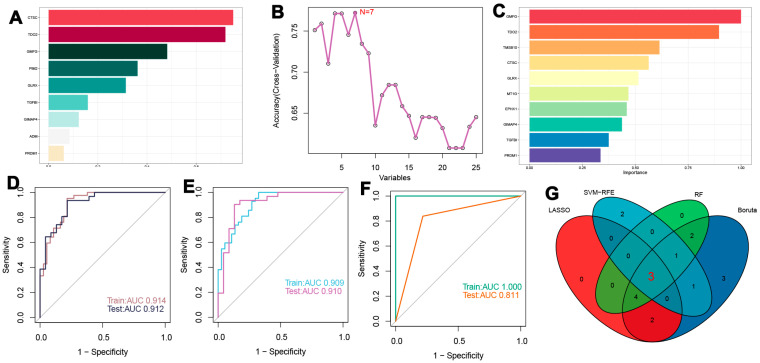
Screening of characteristic genes using machine learning. (**A**) Important genes identified using the LASSO algorithm. (**B**,**C**) Assessment results of SVM and RF models. (**D**) ROC curves of the LASSO model in training and test sets. (**E**,**F**) Predictive performance of SVM-RFE and RF models. (**G**) Characteristic genes for atherosclerosis associated with disulfidptosis.

To identify potential biomarkers for diagnosing atherosclerosis, machine learning techniques were employed to extract key features. This study confirmed the successful correction of batch effects present across the datasets. Following this, the integrated datasets, which included both training and validation sets, underwent additional processing. Initially, genes with non-zero coefficients—specifically CTSC, TDO2, GMFG, PIM2, GLRX, TGFBI, GIMAP4, ADM, and PRDM1—were identified using the LASSO technique (Figure 7A and Figure S3A,B). Subsequently, the Boruta algorithm highlighted 16 significant variables (Figure S3C). The evaluation of these variables was further refined using models such as SVM and RF to enhance the selection process (Figure 7B,C). ROC curve analysis applied to the training dataset demonstrated that the LASSO model achieved an Area Under the Curve (AUC) of 0.914, while the test dataset yielded an AUC of 0.912, indicating excellent predictive performance for atherosclerosis (Figure 7D). The SVM model, which employed Recursive Feature Elimination, along with the RF model, also exhibited similarly high predictive accuracy, achieving significant AUC values across both training and testing datasets (Figure 7E,F). In conclusion, a Venn diagram illustrated TGFBI, GMFG, and CTSC as key genes associated with both atherosclerosis and disulfidptosis (Figure 7G).

### 3.7. Development and Validation of a Risk Score Model for Atherosclerosis Diagnosis

**Figure 8:**
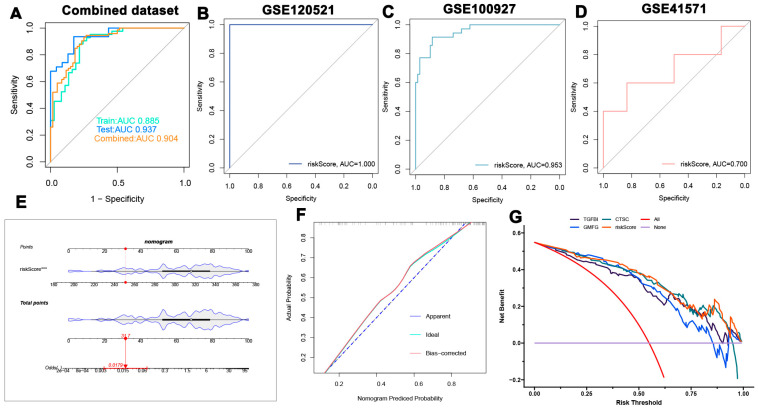
Development and validation of the diagnostic risk score model. (**A**–**D**) ROC curve analysis of the risk score model across different datasets. (**E**,**F**) Calibration curves for the risk score model. (**G**) Decision curve analysis showing potential clinical benefits for patients with atherosclerosis. *** *p* < 0.001.

A risk-scoring model was developed using the formula derived from the coefficients of the LASSO model associated with three specific genes: risk score = (0.159579357 × expression of TGFBI) + (0.482027946 × expression of GMFG) + (0.750202393 × expression of CTSC). ROC analysis conducted on both the combined dataset and individual datasets (GSE120521, GSE100927, and GSE41571) demonstrated high AUC values, all exceeding 0.7. These findings indicate a high level of accuracy for the diagnostic model based on the risk score (Figure 8A–D). Furthermore, a nomogram was created for predicting atherosclerosis using the risk score model, and the calibration curve (Figure 8E,F) confirmed the nomogram’s stability. Patients were categorized into two groups: high-risk and low-risk, based on the median risk score. Additionally, the decision curve analysis (DCA) of the nomogram highlighted its potential clinical benefits for individuals diagnosed with atherosclerosis (Figure 8G).

### 3.8. Pathway Enrichment and Immune Dynamics in High- and Low-Risk Atherosclerosis Groups

**Figure 9:**
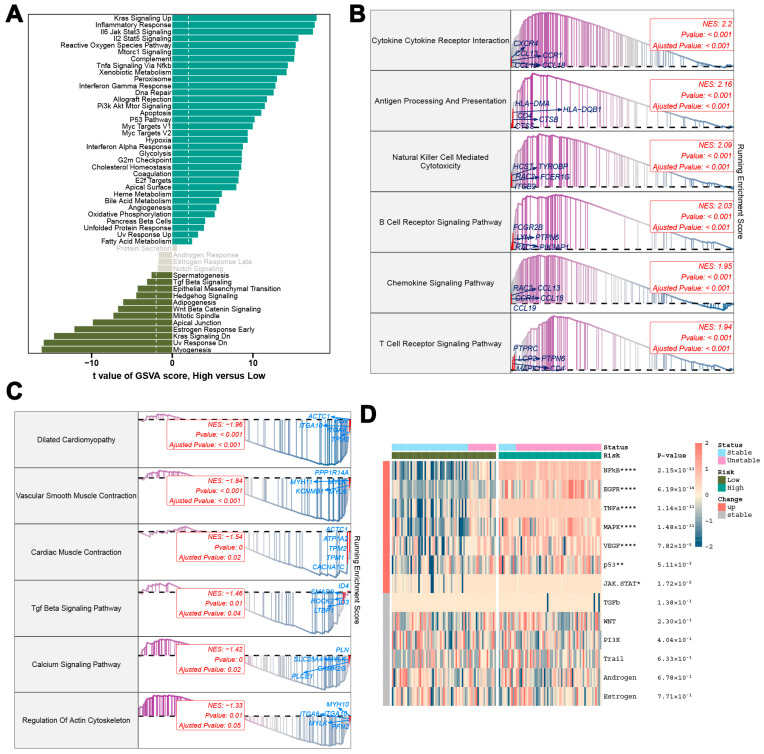
Biological function analysis of characteristic genes in high-risk and low-risk groups. (**A**) Comparison of gene features between high-risk and low-risk groups. (**B**) Upregulated pathways in the high-risk group. (**C**) Downregulated pathways in the high-risk group. (**D**) Comparison of pathway activities between patients with different atherosclerosis risk levels. * *p* < 0.05, ** *p* < 0.01, **** *p* < 0.0001.

To achieve a more comprehensive understanding of the mechanisms involved in atherosclerosis, we conducted an analysis of the feature genes associated with high-risk and low-risk groups. The ten most prominent genes identified were NPNT, PCDH7, HEY2, MYLK, INPP5A, PTAFR, BID, MPP1, C15orf48, and SLC39A8 (Figure S4A,B). Subsequently, biological function annotations and pathway enrichment analyses performed using GSVA and GSEA revealed significant differences between the two groups. The high-risk cohort exhibited notable pathway enrichment associated with critical physiological and pathological states, including cancer development, metabolic abnormalities, immune system responses, and cellular homeostasis. In contrast, the low-risk cohort demonstrated pathways that underscored the complex and interrelated nature of cellular activities, signaling behaviors, and structural components essential for organism development and function (Figure 9A). Further analysis of the high-risk cohort identified the six most significantly upregulated pathways: Cytokine–Cytokine Receptor Interaction, Antigen Processing and Presentation, Natural Killer Cell-Mediated Cytotoxicity, B Cell Receptor Signaling Pathway, Chemokine Signaling Pathway, and T Cell Receptor Signaling Pathway (Figure 9B). Additionally, the six primary pathways that were significantly down-regulated included Dilated Cardiomyopathy, Arrhythmogenic Right Ventricular Cardiomyopathy (ARVC), Hypertrophic Cardiomyopathy (HCM), Vascular Smooth Muscle Contraction, Propanoate Metabolism, and the TGF-Beta Signaling Pathway (Figure 9C). A substantial difference in the activity levels of pathogenic pathways was also observed between high-risk and low-risk patients. Those categorized as high-risk exhibited heightened activity in pathways such as NF-kB, TNFa, MAPK, EGFR, and VEGF, whereas low-risk patients showed upregulation in the PI3K and Androgen pathways (Figure 9D).

### 3.9. Immune Infiltration and Immune Modulator Expression in High- and Low-Risk Groups

**Figure 10:**
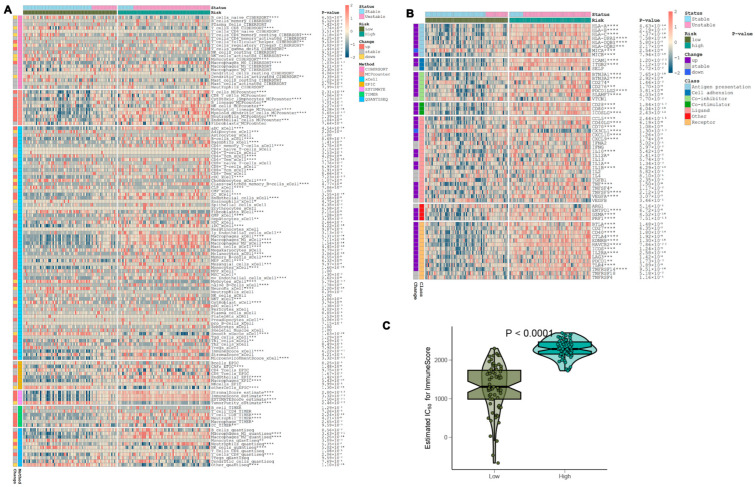
Analysis of immune cell infiltration levels. (**A**) Heatmap of immune cell infiltration in high-risk and low-risk groups. (**B**) Differential expression of immune modulators between risk groups. (**C**) Comparison of immune scores across risk profiles. * *p* < 0.05, ** *p* < 0.01, *** *p* < 0.001, **** *p* < 0.0001.

Levels of immune infiltration were evaluated using algorithms such as ssGSEA, MCPcounter, xCell, ABIS, and ESTIMATE. A heatmap illustrating the patterns of immune cell infiltration in both high-risk and low-risk groups, as well as in stable versus unstable plaque conditions, was created using these algorithms (Figure 10A). Notably, the high-risk group exhibited increased recruitment and activation of immune cells, with samples from this group predominantly associated with an unstable state. To further investigate, an analysis was conducted to compare the expression of immune modulators between the high-risk and low-risk cohorts, considering both stable and unstable plaque conditions. This analysis aimed to enhance the understanding of the immune landscape related to atherosclerosis pathology (Figure 10B). Genes associated with antigen presentation (including HLA-A, B, C, DPA1, DQB1, DQB2, and MICB), cell adhesion (ICAM1, ITGB2, and SELP), immune checkpoints (BTN3A1, BTN3A2, CD274, CD276, PDCD1LG2, and SLAMF7), co-stimulation (CD28, CD80, and ICOSLG), ligands (CCL5, CD40LG, CD70, CXCL10, CXCL9, IL10, IL12A, IL1B, TGFB1, TNF, TNFSF4, TNFSF9, and VEGFA), and receptor expression (BTLA, CD27, CD40, CTLA4, EDNRB, HAVCR2, ICOS, IL2RA, LAG3, PDCD1, TRL4, and TNFRSF14), along with additional genes (ENTPD1, GZMA, and PRF1), demonstrated a significant upregulation in the high-risk cohort (Figure 10B). Furthermore, the immune scores across the various risk groups were quantitatively assessed, providing a comprehensive analysis of their immune profiles. Notably, the high-risk group exhibited substantially elevated immune scores compared to the low-risk group (Figure 10C).

### 3.10. CTSC Knockdown Mitigates Atherosclerosis Progression in In Vitro and In Vivo Models

**Figure 11:**
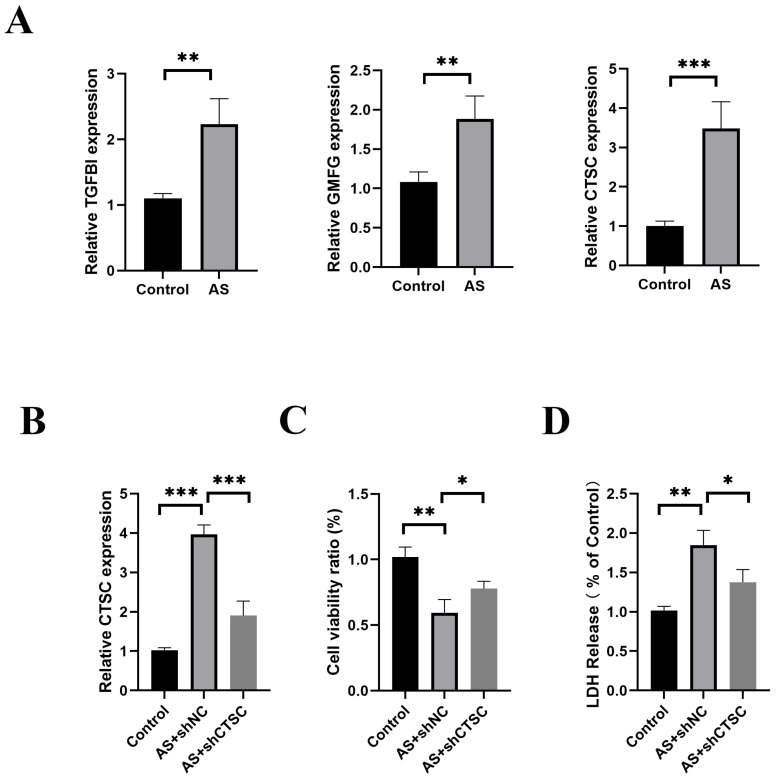
In vitro validation of CTSC knockdown in the SMC-based atherosclerosis model. (**A**) Expression levels of CTSC, TGFBI, and GMFG in the SMC-based in vitro atherosclerosis model. (**B**) Efficiency of lentivirus-mediated CTSC knockdown in the SMC-based atherosclerosis model. (**C**) Effect of CTSC knockdown on cell viability in the SMC-based atherosclerosis model. (**D**) LDH release in SMCs following CTSC knockdown in the atherosclerosis model. * *p* < 0.05, ** *p* < 0.01, *** *p* < 0.00.

**Figure 12:**
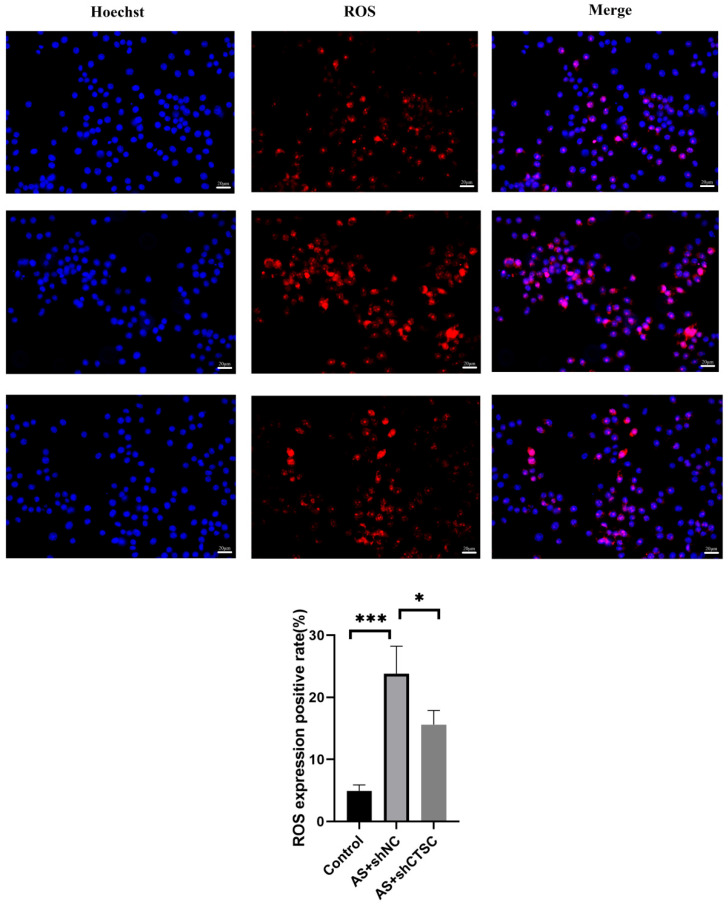
ROS fluorescence detection in SMCs following CTSC knockdown in the atherosclerosis model. * *p* < 0.05, *** *p* < 0.00.

**Figure 13:**
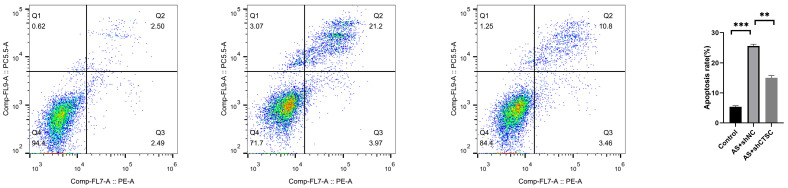
Flow cytometry analysis of apoptosis in SMCs with CTSC knockdown in the atherosclerosis model. ** *p* < 0.01, *** *p* < 0.001.

Models of atherosclerosis were developed both in vivo and in vitro to elucidate the role of the characteristic gene CTSC. In the smooth muscle cell (SMC)-focused in vitro model of atherosclerosis, RT-qPCR analysis demonstrated that the expression levels of CTSC, TGFBI, and GMFG were significantly elevated compared to those in the control group (Figure 11A). These findings are consistent with previous studies, thereby strengthening the validity of establishing a risk prediction model that incorporates these three genes. The effectiveness of lentivirus-mediated CTSC knockdown was confirmed, revealing a greater than 50% reduction in CTSC expression (Figure 11B). Notably, CTSC knockdown significantly enhanced cell viability in the SMC-based atherosclerosis model (Figure 11C) and decreased LDH release (Figure 11D). Furthermore, ROS fluorescence detection indicated that CTSC knockdown led to a reduction in ROS expression levels in the SMC-based atherosclerosis model when compared to the empty viral vector control group (Figure 12). Flow cytometry analysis further illustrated that CTSC knockdown markedly diminished apoptosis in SMCs within the atherosclerosis model (Figure 13). These findings suggest that the reduction in SMC death during the progression and development of atherosclerosis is associated with CTSC knockdown, indicating that CTSC may serve as a critical regulator of disulfidptosis under atherosclerotic conditions.

**Figure 14:**
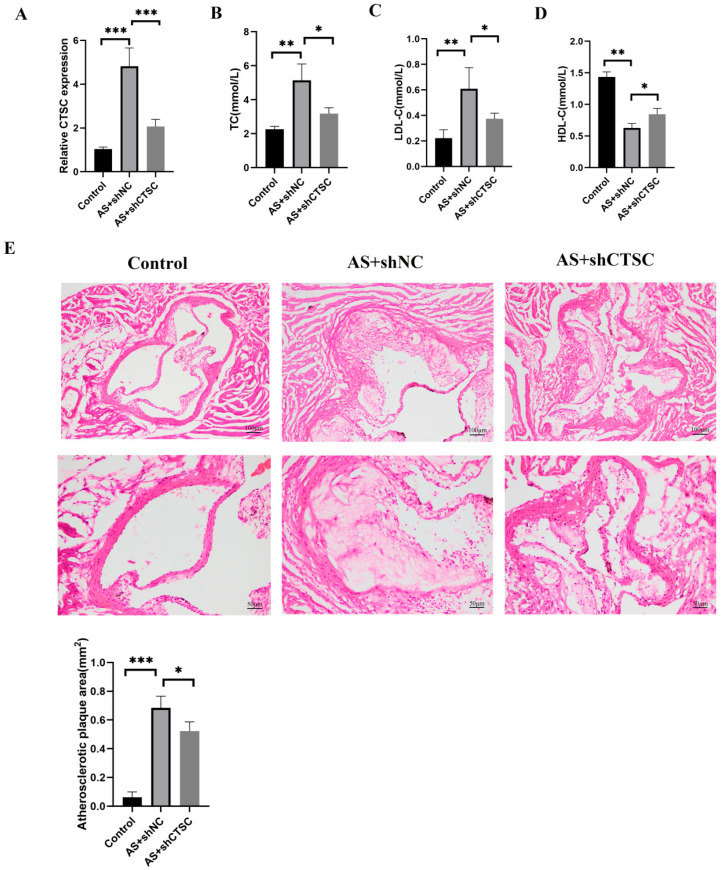
In vivo evaluation of CTSC knockdown in the rat atherosclerosis model. (**A**) PCR confirmation of CTSC knockdown efficiency in the rat atherosclerosis model. (**B**) TC levels in the rat atherosclerosis model following CTSC knockdown. (**C**) LDL-C levels in the rat atherosclerosis model following CTSC knockdown. (**D**) HDL-C levels in the rat atherosclerosis model following CTSC knockdown. (**E**) HE staining showing plaque area reduction following CTSC knockdown in the rat atherosclerosis model. * *p* < 0.05, ** *p* < 0.01, *** *p* < 0.001.

To further investigate the role of CTSC in atherosclerosis, a rat model of atherosclerosis was established. A CTSC knockdown model was created through tail vein injection of AAV vectors, with PCR analysis confirming a knockdown efficiency of over 60% (Figure 14A). Compared to the empty vector group, CTSC knockdown resulted in a significant reduction in TC levels (Figure 14B) and LDL-C levels (Figure 14C), while HDL-C levels were markedly increased (Figure 14D). Hematoxylin and eosin (HE) staining revealed that CTSC knockdown effectively reduced plaque area in the rat atherosclerosis model. These findings suggest that CTSC knockdown mitigates the formation of atherosclerotic plaques.

## 4. Discussion

Atherosclerosis is a complex condition characterized by the involvement of various cell types, including SMCs, endothelial cells, and immune cells [18]. Advances in single-cell genomic technologies are facilitating new research in both mouse models of atherosclerosis and human plaques, thereby illuminating the distinct cellular composition of these lesions [19,20]. The growing body of evidence indicates that the phenotypic transition of SMCs is crucial for the progression of atherosclerotic disease [21,22]. During this transition, SMCs located in the arterial wall undergo processes of proliferation, migration, and differentiation into various cell types within atherosclerotic lesions [23,24,25]. This phenomenon is fundamentally significant to disease progression, lesion stability, and associated clinical complications [26]. Recent advancements in human genetics, coupled with innovative techniques such as single-cell profiling and lineage tracing, have illuminated the significant contributions of SMCs and their derivatives to the cellular phenotypes that regulate atherosclerosis. These findings highlight the complex and multifaceted underpinnings of the disease, emphasizing how SMCs dynamically influence the cellular microenvironment of atherosclerotic plaques. Notably, certain sub-phenotypes of SMCs, referred to as synthetic/dedifferentiated cell (SDC) sub-phenotypes, have been identified as exerting dual modulatory effects on atherosclerotic progression. On the one hand, they can promote lesion stability and slow disease progression; on the other hand, they may contribute to destabilization and exacerbate the clinical condition. This delicate balance in understanding SMC sub-phenotypes necessitates a concerted effort to develop targeted therapeutic strategies that consider the interplay of diverse cellular dynamics in the management of atherosclerosis [27,28]. However, despite these advancements, many important questions remain regarding the specific roles and mechanisms by which SMCs may enact phenotypic modulation in atherosclerosis.

This study utilized scRNA-seq to analyze plaques from both the PA and AC groups. Consistent with prior findings, the predominant cell populations identified included SMCs, endothelial cells, T cells, and macrophages, all of which play significant roles in atherogenesis [19,23]. scRNA-seq has emerged as a powerful tool for unveiling cellular heterogeneity within atherosclerotic plaques, providing unique insights into their complexity. Recent research has demonstrated that various macrophage subsets inhabit atherosclerotic plaques, including classical inflammatory macrophages, foam cell-like macrophages, and TREM2 high-expressing macrophages. These macrophage classes appear to perform specialized functions in lipid metabolism and pathological calcification [29,30]. Additionally, scRNA-seq has begun to elucidate the intricate networks of intercellular communication within atherosclerotic lesions. Notably, it has revealed critical interactions between endothelial cells and immune cells, which seem to surpass the influence of individual contributions in the context of chronic atherosclerosis [31,32]. Furthermore, these interactions may be pivotal in determining relevant pathophysiological processes related to plaque stability, immune activation, and vascular remodeling. Collectively, these findings not only enhance our understanding of the mechanisms underlying atherosclerotic disease but also propose novel molecular and cellular targets that could guide the development of future therapeutic strategies aimed at mitigating disease progression and improving patient outcomes.

Subsequently, four algorithms were employed to analyze the distribution of disulfidptosis across all cell types in patients with atherosclerosis within the combined dataset. Notably, SMCs exhibited the highest density of disulfidptosis across all four methods. Disulfidptosis, a form of cell death induced by disulfiram (DSF), has been investigated for its potential therapeutic applications in various diseases. Numerous studies suggest that disulfidptosis is significantly associated with prognosis and response to immunotherapy in lung adenocarcinoma. The disulfidptosis-related gene model, constructed using machine learning techniques, can effectively predict the survival rates and treatment responses of lung adenocarcinoma patients [33,34]. Additionally, research on disulfidptosis in acute myeloid leukemia has shown that the upregulation of its associated genes is correlated with poor prognosis. Increased disulfidptosis activity scores are associated with worse clinical outcomes and an immunosuppressive state [35]. Although the role of disulfidptosis in atherosclerosis remains underexplored, investigations into disulfidptosis-related genes in bladder cancer underscore their contributions to tumor development, treatment responsiveness, and patient outcomes. This research suggests that POU5F1 and CTSE may serve as promising therapeutic targets [36]. DSF shows potential as a novel adjuvant therapy for atherosclerosis by concurrently modulating multiple atheroprotective pathways, including the inhibition of GsdmD, reduction in inflammatory markers, induction of autophagy, enhancement of efferocytosis and phagocytosis, and beneficial modulation of gut microbiota [10]. However, the mechanisms by which disulfidptosis operates in atherosclerosis have yet to be examined. This research highlights significant relationships between SMCs and various immune cells, including macrophages, T cells, and neutrophils, in both groups, emphasizing the complex communication that influences plaque stability or instability. Intercellular communication analysis revealed unique interaction networks, with SMCs functioning as a hub for signaling in both stable and unstable regions. Notably, this study extends previous findings by detailing distinct pathways activated in different regions. For instance, the pro-inflammatory CCL and CXCL pathways were prominent in stable regions, while extracellular matrix-related pathways, such as SPP1 and GALECTIN, were enriched in unstable regions. Research has similarly demonstrated that the CCL and CXCL families of chemokines are crucial for recruiting and activating immune cells, which, in turn, facilitate inflammatory responses and lesion development in atherosclerosis [37,38]. Among individuals suffering from atherosclerosis, CCL5 is recognized as a potentially crucial factor in the reprogramming of myeloid cells. In the plasma of patients, CCL5 facilitates specific signaling roles in innate immune cells, which serve as markers of inflammation associated with atherosclerotic conditions [39]. Oxidation-modified low-density lipoprotein (ox-LDL) promotes atherosclerosis through the release of CXCL1, a chemokine that is anchored to the surface of endothelial cells, facilitating monocyte adhesion and the progression of atherosclerosis. Numerous studies have demonstrated that the inhibition of lysophosphatidic acid receptors reduces the retention of arterial leukocytes and the progression of atherosclerosis due to hyperlipidemia [40]. SPP1 and galectin, along with other molecules, play key roles in the formation and remodeling of the extracellular matrix, which in turn influences plaque stability [41]. This insight underscores the variations in cellular signaling factors across different regions and their profound impact on plaque stability and disease progression.

Through this pseudotime-based trajectory analysis, we have gained insights into the development of smooth muscle cells in atherosclerotic lesions. The distinct activation patterns observed between SMC whole-cell populations characterized by varying levels of disulfidrosis highlight their temporal and functional heterogeneity. In the disulfidrosis-rich group, progressive activation over time supports its involvement in chronic inflammation and extracellular matrix remodeling. Conversely, the biphasic activation pattern in disulfidrosis-poor SMCs indicates their dynamic responsiveness to environmental factors. Thus, these contrasting activation trends provide a foundation for future studies to assess the relationship between temporal SMC activation, plaque stability, and patient outcomes while also identifying potential targets for therapeutic intervention.

Machine learning-based techniques, including LASSO, Boruta, Support Vector Machine, and Random Forest, were employed to specifically identify CTSC, TGFBI, and GMFG as potential diagnostic biomarkers associated with SMC activity in atherosclerosis. Among these, CTSC encodes the enzyme Cathepsin C, a lysosomal cysteine protease that is crucial for the functionality of immune cells. Cathepsin C is essential for the activation of various serine proteases involved in immune responses. This regulatory role underscores the enzyme’s significance in modulating the immune system and in a cascade of complex immune mechanisms [42]. Although the specific contribution of CTSC to atherosclerosis remains unclear at present, insights from its role in the tumor microenvironment provide valuable direction. For instance, CTSC can facilitate the seeding of metastatic breast cancer to the lungs by mediating neutrophil infiltration and the formation of neutrophil extracellular traps, which are implicated in breast cancer dissemination [43,44]. Similarly, evidence suggests that its involvement may also influence inflammatory pathways and cell migration processes in atherosclerosis; however, further investigation is required to elucidate its role within the mechanisms underlying this condition.

The gene TGFBI encodes a protein that plays a crucial role in various physiological processes, including cell growth, differentiation, migration, and apoptosis. TGFBI is particularly important in the remodeling of the extracellular matrix and in tissue repair [45]. This gene is closely associated with SMC function and differentiation. For example, MEOX1, a novel regulator of SMC differentiation induced by TGF-β, operates through the PI3 kinase and Smad3 signaling pathways—both of which also drive TGFBI expression [46]. Although direct evidence linking TGFBI to atherosclerosis is limited, its interaction with the extracellular matrix implies a potential role in the progression of the disease. Specifically, TGFBI’s capacity to influence extracellular matrix composition and cellular dynamics may impact plaque stability and vascular remodeling.

A risk score model concerning SMCs and a corresponding nomogram were developed based on unique characteristic genes, demonstrating high diagnostic efficiency. The assessment of immune infiltration and the expression of immune-modulatory genes in both high- and low-risk groups provided valuable immunological insights regarding atherosclerosis. Enhanced immune activation and the expression of modulators in high-risk participants reveal the primary inflammatory milieu associated with plaque instability and development. The identification of CTSC as a critical regulator of SMC demise and plaque development underscores its significance for therapeutic approaches. Both experimental (in vitro) and organismal (in vivo) models indicated that reducing CTSC levels alleviates oxidative stress, decreases apoptosis, and diminishes plaque size. These improvements were associated with better lipid profiles, highlighting the overarching benefits of targeting CTSC. At the molecular level, the reduction in CTSC was found to decrease disulfidptosis and enhance cell survival, suggesting a protective effect against SMC loss within plaques. These results provide a compelling basis for developing therapeutics aimed at CTSC to prevent plaque destabilization and mitigate atherosclerotic consequences.

Despite the strength of these findings, certain caveats warrant examination. The reliance on existing datasets may introduce biases related to sample selection and processing. Future research should aim to validate these findings across multiple cohorts, including longitudinal analyses to capture temporal changes in plaque biology. Additionally, functional tests are necessary to elucidate the causal involvement of the identified genes and pathways in vivo. The clinical applicability of the diagnostic approach requires further confirmation through prospective investigations, particularly in diverse populations.

This study provides a comprehensive multi-dimensional analysis of the cellular and molecular environment in atherosclerosis, offering unique insights into immune cell interactions, signaling pathways, and potential biomarkers. Future research is essential to validate these findings and to investigate treatment strategies that target the newly identified pathways and biomarkers.

## 5. Conclusions

This study identifies disulfidptosis as a critical mechanism in atherosclerosis progression, with SMCs exhibiting the highest disulfidptosis activity. Through single-cell RNA sequencing and machine learning, we pinpointed CTSC, TGFBI, and GMFG as key biomarkers for atherosclerosis risk stratification. Experimental validation demonstrated that CTSC knockdown reduces oxidative stress, inhibits SMC apoptosis, and mitigates plaque burden in vitro and in vivo. These findings highlight CTSC as a promising therapeutic target for stabilizing atherosclerotic plaques. Future studies should explore longitudinal changes in disulfidptosis dynamics and validate the diagnostic model in diverse patient cohorts.

## Data Availability

The original contributions presented in the study are included in the article and Supplementary Material, further inquiries can be directed to the corresponding author.

## Supplementary Materials

The following supporting information can be downloaded at: https://www.mdpi.com/article/10.3390/biomedicines13040926/s1, Figure S1: Immune cell marker expression in atherosclerotic plaques; Figure S2: Phenotypic analysis of disulfidptosis-rich and disulfidptosis-poor SMCs; Figure S3: Machine learning-based identification of characteristic genes. (A) LASSO regression identifies key genes for atherosclerosis. (B) Boruta analysis highlights significant variables. (C) Venn diagram showing CTSC, TGFBI, and GMFG as final selected genes from multiple models; Figure S4. Pathway enrichment analysis in high- and low-risk groups. (A) Key genes distinguishing high-risk and low-risk groups. (B) Pathways enrichment in high-risk and low-risk groups.
