# Supplementary material for: Immune Regulation and Disulfidptosis in Atherosclerosis Influence Disease Progression and Therapy
Source: Biomedicines. 2025 Apr 9;13(4):926. doi: 10.3390/biomedicines13040926 (PMC12025079; doi:10.3390/biomedicines13040926)
Supplement: Supplementary file 1 [file biomedicines-13-00926-s001.zip › biomedicines-3498660-supplementary.pdf]

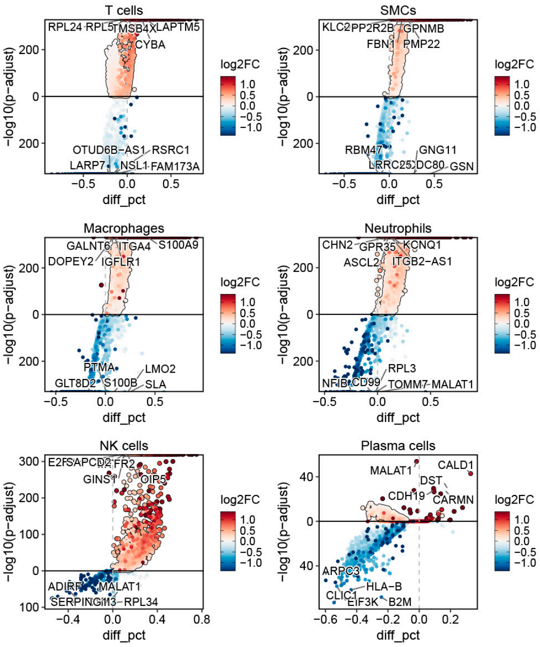

Figure S1. Immune cell marker expression in atherosclerotic plaques.

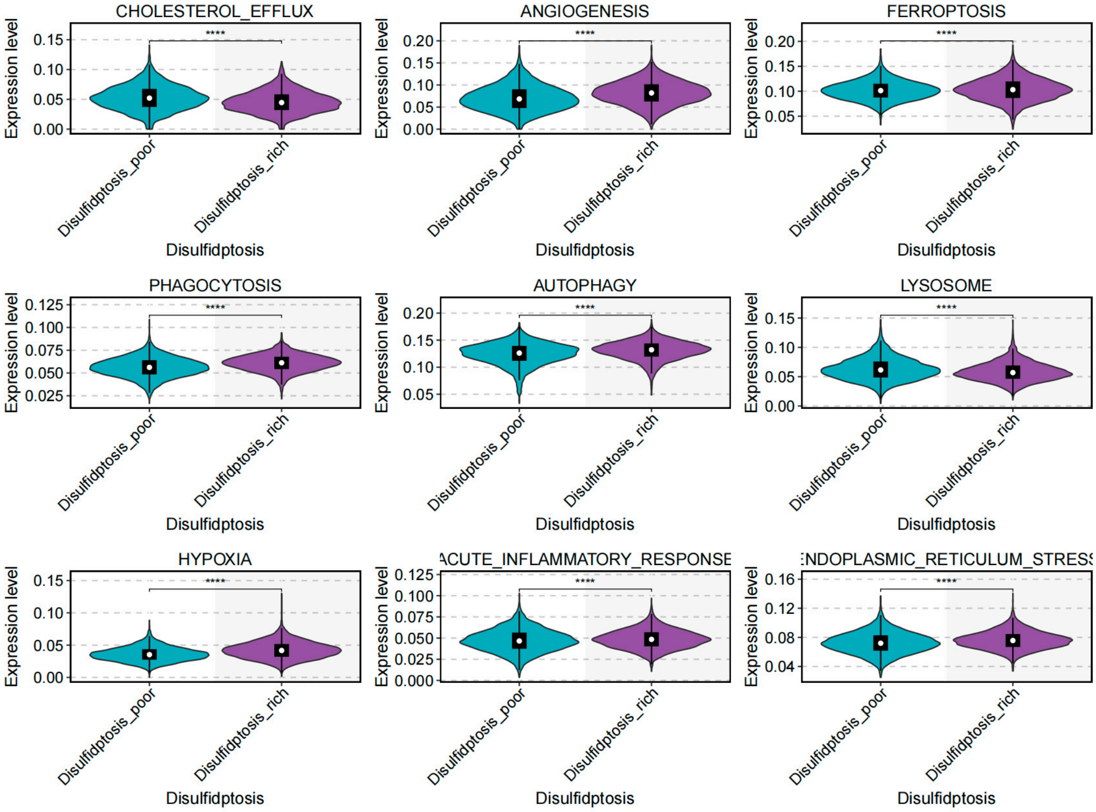

Figure S2. Phenotypic analysis of disulfidptosis-rich and disulfidptosis-poor SMCs.

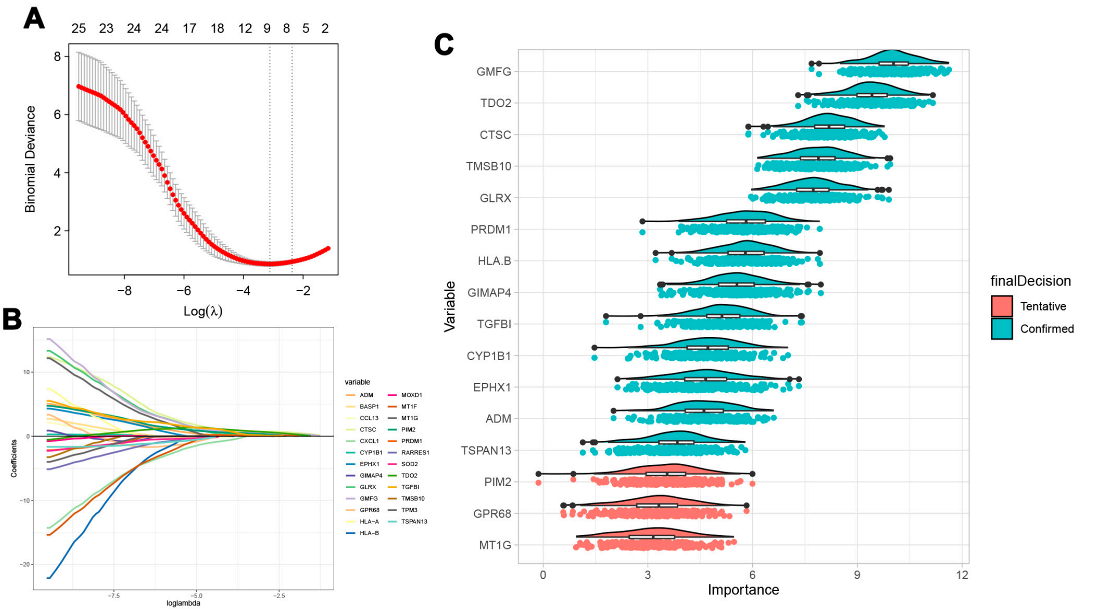

Figure S3. Machine learning-based identification of characteristic genes. (A) LASSO regression identifies key genes for atherosclerosis. (B) Boruta analysis highlights significant variables. (C) Venn diagram showing CTSC, TGFBI, and GMFG as final selected genes from multiple models.

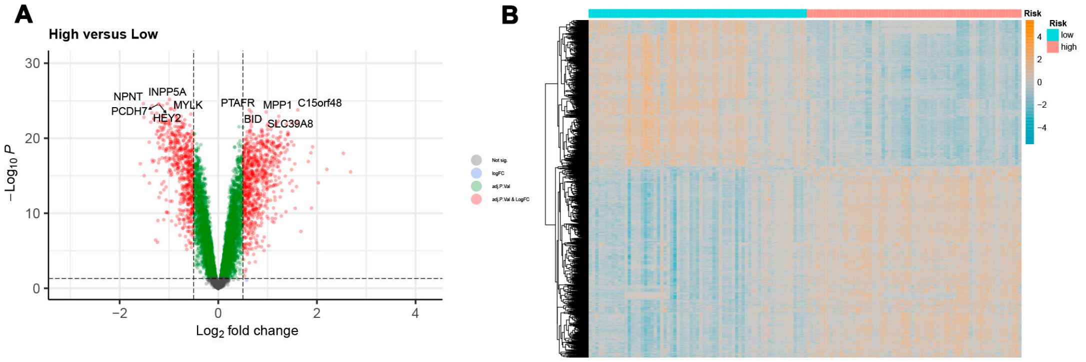

Figure S4. Pathway enrichment analysis in high- and low-risk groups. (A) Key genes distinguishing high-risk and low-risk groups. (B) Pathways enrichment in high-risk and low-risk groups.
